# A Study of Changing Automated Teller Machine (ATM) Accessibility and its Implications in England and Wales

**DOI:** 10.1007/s12061-025-09658-2

**Published:** 2025-05-21

**Authors:** Stephen D. Clark, Chris Duley

**Affiliations:** 1 School of Geography and Consumer Data Research Centre, University of Leeds, Leeds, LS2 9 JT UK; 2 Consumer Data Research Centre, University of Leeds, Leeds, LS2 9 JT UK

**Keywords:** Accessibility, Automated Teller Machines (ATMs), England and Wales, Deprivation

## Abstract

The ways that people choose to pay for products and services is changing, with many people having a greater desire to pay using electronic means in preference to using cash. However for a significant section of society cash remains an important option, for budgetary, technical and sociological reasons. One of the primary ways to make cash available is through Automated Teller Machines (ATMs) and the United Kingdom’s financial industry and the government are keen to maintain a viable network of ATMs. In this study a refinement to the Floating Catchment Area technique, the Modified Huff Variable Three Step Floating Catchment Area (MHV3SFCA), is used to assess the accessibility of ATMs in England and Wales. The MHV3SFCA approach better accounts for competition, and ensures equity through a minimum threshold for access. How this accessibility has changed over time and how the definition of the network has an impact is shown. The results are illustrated with maps that identify potential ATM deserts and a case study of the City of York. Trends are summarised using the typographies of an area classification, a deprivation index and an urban/rural indicator. The results show better accessibility in deprived and urban locations. Over a 3 year time period the accessibility has deteriorated for all locations, however this was least in deprived areas, where accessibility is already good. In locations where there has been an attempt to protect ATMs, the level of accessibility has remained stable.

## Introduction

The changes in banking technology over the past decade has caused a shift towards an increase in the use of electronic payments for goods and services, particularly through contactless card payments (Hall et al., 2022). These changes were greatly accelerated by the COVID-19 pandemic, where individuals had problems physically travelling to locations to obtain banking services, withdraw cash from banks, or purchase goods at physical stores (Litt, 2021; Nanda et al., 2021; Sonea et al., 2019).

However, there are increasing concerns in the United Kingdom (UK) that this shift has gone too far, with there being issues around the ability to access cash in order to purchase goods and pay for services (Ceeney, 2019), particularly for those individuals at the ‘financial fringe’ (Collard et al., 2016; Appleyard et al., 2023). The Financial Conduct Authorities’ Financial Lives 2020 survey (Financial Conduct Authority, 2023) found that 5.4 million adults said they relied on cash to a great or very great extent, and that this reliance was highest (42%) among adults aged 85 +. In evidence to the House of Commons Welsh Select Committee the Automated Teller Machines (ATM) operator NoteMachine Ltd stated that “*the UK [is] facing both a cliff-edge in free cash access, undermining payment choice for businesses and severing a lifeline for vulnerable consumers*” (Welsh Affairs Committee, 2024). A Financial Conduct Authority (2021) study identified a typology of six types of vulnerable individuals who used cash for the majority of their everyday purchases or said they would struggle or be unable to cope in a cashless society. This desire to use cash was driven by a number of mechanisms: financial exclusion; behavioural barriers; lack of capability; previous life events; and a wish to ‘defend’ cash. The most vulnerable to the absence of cash were ‘Needs-based Users’ who had no other choice than to use cash, being excluded from other financial alternatives. The next most vulnerable were the ‘Functional Users’ who possessed low financial resilience and used cash as a mechanism to control their budget (Jonker, 2016). With moderate vulnerability, there were the ‘Older & Disengaged’ types who are typically older with established cash-based habits and with a technological barrier to use alternatives to cash (Cooper, 2016). The remaining types were not so vulnerable and have more of a desire to use cash as a ‘lifestyle choice’. These are the ‘Cash Defenders’ (they want to preserve cash as an option), the ‘Comfortable and Capable’ (they use some on-line services but are not adverse to the use of cash) and ‘Impulse Avoiders’ (who use cash to manage their behaviour traits).

Whilst typically this desire to use cash was confined to certain demographics or social groups as typified by the Financial Conduct Authority (2021) report, there are more recent reports of a more widespread desire for households wanting to use cash, both as a mechanism to budget effectively during a ‘cost of living crisis’ (Noor Nanji & Henshall, 2022) and make certain types of payment, e.g. personal payments/tipping, small grocery shopping or paying for food or drink when socialising (Welsh Affairs Committee, 2024).

One impact of this shift to electronic payments is that there has been a rapid decrease in the number of retail bank branches (Panjwani et al., 2024). These branches were one of the primary places to obtain cash (and other financial services), so their loss is keenly felt in local neighbourhoods. Within the UK there are however two other main sources of cash, Post Office branches and ATMs.

Prior to 2010 the network of Post Offices was rationalised, with a gradual reduction in the number of branches from around 21,000 in 1986 to 12,000 in 2010. Since 2010 however the number of Post Offices has remained relatively constant (Booth, 2023) and there is now an obligation on the Post Office to ensure that everyone has good access to its network though six ‘Access Criteria’ metrics (House of Commons, 2019, Sect. 3.0) and that it can offer everyone a basic ‘Universal banking’ service (Midgley, 2005; Sonea & Westerholt, 2021).

More widely, the availability of ATMs has also been another area where policies have been developed to try and maintain access to cash. ATMs can be located within a premises, either a bank, Post Office or convenience store (30% of free to use ATMs are located in branches (LINK, 2024a)), or externally, via a ‘hole in the wall’ locations.

Other options for cash are through the provision of counter terminals or ‘cash back’. Counter terminals are primarily located within convenience stores, where a machine can verify the identity of the card holder and cash is dispensed from the shops till. ‘Cash back’ can also be provided by the retailer, again after having verified the identity of the card holder. Obviously, in store ATMs, counter terminals and cash back are only available during opening hours whilst the external locations are available 24/7.

Like with the Post Office network, the ATM network has shown recent significant reductions in the number of ATM locations. According to the LINK organisation (which manages the UK ATM network), between January 2018 and February 2024, the number of free-to-use ATMs has reduced from 54,500 to 36,500 (LINK, 2022). These reductions have been most keenly felt in those ATMs located in branches, with data showing a 9% reduction in ATMs located in branches between 2022 and 2023, compared with a smaller 4% reduction in non-branch locations (LINK, 2024a). To help regulate this reduction, LINK has attempted to ensure that if a free-to-use ATM is closed, or switches to surcharging the customer, it will look to provide alternative or equivalent access for the affected neighbourhood. The organisation also identifies (on request) neighbourhoods at serious disadvantage if they were to be without access to an ATM (LINK, 2024b) through its Financial Inclusion Program (FIP) and incentivises operators to maintain ATMs in such neighbourhoods by changing the fee structure it pays.

The motivation for this study is to examine how accessible the current ATM network is for the general population. This has not previously been done for this specific service. England and Wales is chosen as the study area since it covers a variety of area types and has consistent population estimates. A variant of the Floating Catchment Area (FCA) technique is used to derive an accessibility score that takes account of potential utilisation of ATMs and ensures equity in the opportunities for access. The study also measures how access to cash has changed in recent years and what the impact of the availability of surcharging ATMs is.

## Literature

Penchansky and Thomas (1981) describe five dimensions for access, with three being aspatial, ‘affordability’, ‘acceptability’, and ‘accommodation’, which all touch on socio-economic aspects, whilst two, ‘availability’ and ‘accessibility’, are spatial. A later consideration of the concept of accessibility and its measurement are provide by Handy and Niemeier (1997), where they emphasise the importance of the problem specification, the calibration and the interpretability of the measure. This broader consideration of access beyond physical access is considered by van Wee (2022) who advocates taking into account more the nature of the population. These include the socio-demographic nature of the population, its attitudes, tolerance of differing levels of access, and flexibility/capability in adapting to different circumstances. By taking account of all these aspects, it is possible to judge the acceptability of access for a population. Geurs and van Wee (2004) also provide a review of the mechanisms of access measures, and highlight the importance of the theoretical basis of the measure, its interpretability, the ease at which it can be used to inform outcomes, and the complexity of the data requirements.

In published studies there are two main approaches that have been used that satisfy most of these criteria. A container approach measures availability of services in a neighbourhood and is usually expressed as a population to provider ratio, e.g. general medical practitioner per person (Moles et al., 2001). This measure is easy to comprehend and is appropriate where the provider and population are restricted to specific areas. It is less appropriate where there is choice, both on the part of the provider and the user. It is also subject to the modifiable areal unit problem (Openshaw, 1984) where the measure is influenced by the drawing of potentially arbitrary boundaries. An alternative approach is to count how many providers are within a radius of the population (cumulative opportunity) (Jo et al., 2020) or how far away services are located (spatial proximity) (Stix, 2020). These measures are again easy to interpret and do not have the same issues as container approaches. They do not however account for competition for the service; they require an external assessment of what a credible value is for the radius or number of services to access; and a choice as to whether the radius is measured as a distance or a time.

### Catchment Studies

The distance band catchment approach was used by Tischer et al., (2020) to examine the geographies of access to cash throughout the UK. Cash access locations were defined as free-to-use ATMs, surcharge ATMs, Cashback locations, Post Offices and retail bank branches. Accessibility was measured by the percentage of the 2011 Census population with an access point within various distance bands ranging from 250 m to 10 km. The closest band that had an access point was also determined. Neighbourhood were categorised into eight groups, from good ‘Close and abundant’ access through to poor ‘Very remote’ access. This work followed on from two earlier case studies, one in Bristol, South West England (Tischer et al., 2019) and South Wales (Evans et al., 2020). These two case studies computed an Availability of Cash (AvCash) index that was built from a weighted sum of the number of different types of cash access points within 1 km of each neighbourhood (and this approach was transferred to a Spanish context by Náñez Alonso et al., (2020)). An interesting study was conducted by Sonea et al, (2019) that looked at access to banking services from a joint physical and a digital perspective. They were particularly interested in whether it was possible to identify physical and digital banking deserts (or ‘The Voids’ in the terminology they used, adopted from Rowe et al., 2015) by exploring the distances to the first and second closest bank branches and combining this with local mobile/fixed internet speeds, and a category within the 2014 version of the Internet User Classification (Consumer Data Research Centre, 2018). They identified that between 63 and 67 thousand people in the UK live in locations that could be considered banking deserts by virtue of poor accessibility.

Another study by Sonea and Westerholt (2021) outlines the six access criteria against which the accessibility of the Post Office network is judged, all are based on the percentage of the population that lies within predefined distance or time thresholds of a Post Office. For the case study of Wales, they evaluate the effectiveness of these criteria if journey time (rather than distance) and mode of travel is used. They also introduce the concept of the capacity of each Post Office, expressed through its opening hours and introduce a spatial hotspot statistic that shows where this capacity is concentrated or is thinly available. Confirming earlier studies (Comber et al., 2009 and Langford & Higgs, 2010) they judged that measured against the Post Office’s own access criteria with a distance metric, its obligations are not always being met, with their estimate being that 3% to 4% of the Welsh population (125,000 people) being underserved.

### Floating Catchment Areas

In the recent literature the concept of a FCA has been proposed as an alternative to these approaches, with the Two Step Floating Catchment Area (2SFCA) introduced by Luo and Wang (2003), being the first of a series of papers that progressively developed the sophistication of this spatial analysis technique. (Jörg & Haldimann, 2023; Subal et al., 2021). The first step of a 2SFCA calculates for each provider, a provider to population ratio within a given buffer catchment, this measures the level of service the provider is realistically able to provide. The second step is to sum these levels of services that lie within a buffer catchment of the population, which is the total service that all the providers are able to supply to that population. The main advantages of 2SFCA over container and catchment approaches are that firstly the results are not sensitive to rigid and potentially irrelevant administrative boarders. Secondly potential competition between locations in terms of accessing the supply is accounted for. Thirdly there is the consideration of multiple supply options rather than a fixed number, allowing potentially all supply locations to satisfy demand. The calculation provides a spatial accessibility index (SPAI, Jörg and Haldimann, 2023) for a location that has the properties that it increases: with a higher number and/or higher capacity of supply locations; with a lower level of competition for the supply; and the closer the supply and demand locations are.

The technique has been most widely applied with access to health care (Langford et al., 2016; Subal et al., 2021; Wan et al., 2012), but has also been used for access to transit (Langford et al., 2012), urban parks (Hu et al., 2020) and fire cover (Kiran et al., 2020). In particular, a recent study in this journal by Langford et al, (2021) establishes the justification for the use of such methods in evaluating the accessibility to financial services, in their case, retail bank branches in Wales. A similar team of authors provide a comparison between using three traditional measures of accessibility (containers, proximity and abundance) and a 2SFCA approach to measure accessibility to General Practice doctor surgeries in Wales, highlighting that only an FCA approach is able to take a joint account the location, ease of travel and competition for services in its calculation (Page et al., 2018). Pardue and Shelton (2024) further illustrate these advantages in the context of access to traditional and alternative financial institutions in Atlanta, Georgia, USA, finding that of the five approaches for measuring accessibility the FCA approach provided “… *a clear visual connection between racial composition, median income and financial access…*”.

## Methods and Data

### Methods

The method of analysis used here is the Modified Huff Variable Three Step Floating Catchment Area (MHV3SFCA) (Jörg & Haldimann, 2023) variant of FCA. Previously, Langford et al., (2021) provided a justification for the use of an Enhanced (Variable) 2 Step FCA (EV2SFCA with variable catchments) and the MHV3SFCA builds on this with a number of refinements, according to Table 3 of Hauser (2023). Firstly through the Huff probabilities in step 1 (see below) it accounts for supply competition, reflecting the idea that the supply of a good or service is not infinite (Huff, 1963). Secondly the measure of accessibility calculated reflects absolute distances rather than relative distances. With relative distances if all service locations increase by a common factor, the accessibility will be unaffected, whilst in reality as any or all locations are further away, their accessibility should decrease. Thirdly MHV3SFCA maintains the demand population as a fixed value, so that the total system demand does not vary as the number of supply locations or the distances vary (see Table 2 of Jörg & Haldimann, 2023). Finally, it is in line with the Variable Catchments (Adaptive) approach in Langford et al., (2021), where a fixed number of bank branch locations are considered for each neighbourhood. The requirement here is to be able to reach at least Q ATM locations, which allows the accessibility of all neighbourhoods to be judged against the same standard and accords with van Wee’s (2022) notion that people should have a minimum level of access. It also possess Geurs and van Wee’s (2004) theoretical requirement through its interpretability, the ease at which it can be used to inform outcomes, and the simplicity of its data requirements. It should be noted that since each ATM location is defined by its unit postcode (Office for National Statistics, 2024a), each location may contain more than one ATM.

The three steps in the calculation of the MHV3SFCA SPAI are described here:

Step 1: Calculation of Huff selection probabilities (Huff_ij_)

1 $${Huff}_{ij}= \frac{{S}_{j}f({t}_{ij})}{{\sum }_{j \in \left\{{t}_{ij}\le {t}_{i}^{rel}\right\}}{S}_{j}f({t}_{ij})} {\varvec{I}}({t}_{ij}\le {t}_{i}^{rel})$$

where i are the demand (population) centres;

j are the service (ATM) locations;

S_j_ is the service capacity at location j, a count of ATMs at the location;

f(t_ij_) is the time decay weight function (see (4b) below);

t_ij_ is the quickest journey time from i to j, by either by walking or by car;

t_i_^rel^ is the relevant catchment to access Q ATM locations;

I(t_ij_ ≤ t_i_^rel^) is a binary indicator to denote if the journey time is less than t_i_^rel^.

Step 2: Calculation of supply ratios for each ATM (R_j_)

2 $${R}_{j}= \frac{{S}_{j}}{\sum_{i\in ({t}_{ij} \le {t}_{max})}{Huff}_{ij}{P}_{i}}$$

where P_i_ is the demand (population) at centre.

Step 3: Calculation of spatial accessibility index (SPAI_i_)

3 $${SPAI}_{i}= \sum_{j \in \left\{{t}_{ij \le {t}_{max}}\right)}{Huff}_{ij}{R}_{j}f({t}_{ij})$$

To operationalise this method some assumptions and parameters are required. Here a Gaussian time decay weight function is used, in preference to the linear function in Langford et al., (2021).

4 $$b=\frac{{-({t}_{max})}^{2}}{\text{ln}(f\left({t}_{max}\right))}$$

and

5 $$f\left({t}_{ij}\right)= {e}^{\frac{-{t}_{ij}}{b}}$$

To define the shape of this function, two parameters are required, the maximum reasonable travel time (t_max_) and the weight at this time f(t_max_). Here a maximum reasonable travel time of 20 min is used and the weight at this time is 0.01. However the choice of these two parameters is less significant in the case of MHV3SFCA than some other FCA approaches. This is because the weights to access only the Q closest locations are used.. Langford et al., (2021) experimented with various values for this value of Q of between 2 and 6, reasoning that people would at most consider 6 retail bank branch locations in their vicinity rather than the tens or hundreds sometimes assumed in health related studies. Here a value greater than 6 should perhaps be used, to reflect the situation that people would expect more choice in ATM locations than bank branch locations, but not too much more. To help in this election for Q, use is made of UK government guidelines that in urban areas people should have an ATM with 1.6 km, and in rural locations within 4.8 km (HM Treasury, 2022). Counting the number of ATM locations within each of these neighbourhood specific buffers shows that 10 is the median number of ATM locations. This leads us to adopt a value of 10 for the value of Q. All calculations are performed within the R programming language (R Core Team, 2022) and use code provided in Hauser (2023).

MHV3SFCA provides a measure of accessibility that has the properties of a SPAI, and a higher SPAI value indicates better access to ATMs. In particular, locations with more ATMs relative to their floating catchment population (R_j_) will be deemed to be more accessible since there is reduced ‘competition’ for their use and there is some redundancy against an ATM being out of use.

ATM deserts can be defined as those neighbourhoods that fall below a certain threshold value, or are the 0.5% of neighbourhoods with the lowest SPAI, which accords with Handy and Niemeier’s (1997) concept of relative accessibility. This later concept is our definition of an ATM desert (the 0.5% lowest). To further illustrate this measure a map is provided of the City of York that shows the accessibility of free-to-use ATM locations in January 2024. On a more general basis, the SPAI is profiled for certain typographies of neighbourhood, a new 2021 Census based area classification (Wyszomierski et al., 2024), a 2011 urban/rural classification (Office for National Statistics, 2016) and a 2019 multiple deprivation index (IMD) (Ministry of Housing Communities & Local Government, 2019).

The free to use June 2024 ATM network is taken as our base case. To illustrate how the reduction and re-distribution of ATMs over the past 3 years has had an impact on accessibility, the SPAIs are re-calculated using the free to use June 2021 network. Additionally the impact of including those ATMs that charge a fee is captured by calculating the SPAIs for a June 2024 network of all ATMs. Finally, LINK has identified a number of neighbourhoods as part of its FIP and the accessibility of ATMs for these 363 neighbourhoods is used to measure how effective this programme has been in maintaining access for such neighbourhoods.

### Data

The location of ATMs is published every 6 months by LINK (LINK, 2024c). This information contains the street name, the unit post code, whether the ATM is free-to-use or surcharges via a fee and also if it is a counter terminal rather than an ATM. In June 2024 there were 41,681 ATMs available in England and Wales at 29,698 locations, with 33,122 being free-to-use (at 22,785 locations) and 8,559 charging a fee (at 7,756 locations). These figures include 1,917 counter terminals. This study also includes an additional 54 ATMs located within Scotland, along the Scottish border since people living in the neighbouring areas of England are able to access them. The single ATM located on the Isles of Scilly, off the west coast of Cornwall is not included. For analysis purposes the supply locations are the centroids of the ATMs unit postcode, and the number of ATMs within each unit postcode are the supply values S_j_ used in Eqs. 1 and 2.

Similar to how Langford et al., (2021) defined the demand for banking services in Wales, the demand for ATM use here is taken as the population aged 16 and older estimated for Mid-2022 (Office for National Statistics, 2024b). In this study we adopt the geography of output area (OA) as our neighbourhood, with there being an average of 260 people aged 16 and over in each OA (Office for National Statistics, 2024c). This demand is located at the population weighted centroid (PWC) of the OA, with the nine OAs on the Isles of Scilly removed (Office for National Statistics, 2024 d).

The travel times between ATMs locations and the PWC are calculated using either the shortest travel time by car (Giraud, 2022) or by walking (assuming a walking speed of 4.8 km/hr), using whichever is the quickest (the two travel modes considered by Sonea & Westerholt, 2021). The car travel times are based on road type specific speed limits, with additional time penalties for turn movements and parking, and best represents uncongested driving conditions. The walking option is particularly relevant for town and city centre ATM locations where vehicular restrictions (one-way systems or bans) artificially inflate car travel times relative to the walk times. To reduce the number of travel time calculations only journey times between ATMs and PWC pairs within a set radius of each other are calculated. This radius depends on urban/sub-urban/rural nature of the OA, and varies from 9.6 km in urban areas to 32 km in rural locations (recall that the Government guidance is for an ATM within 1.6 km in urban areas, and in rural locations within 4.8 km, but here much more generous thresholds are applied). For all other pairs the journey time is set at 2 h..

## Results

In this section the values of the SPAI are illustrated.

### Maps

**Fig. 1:**
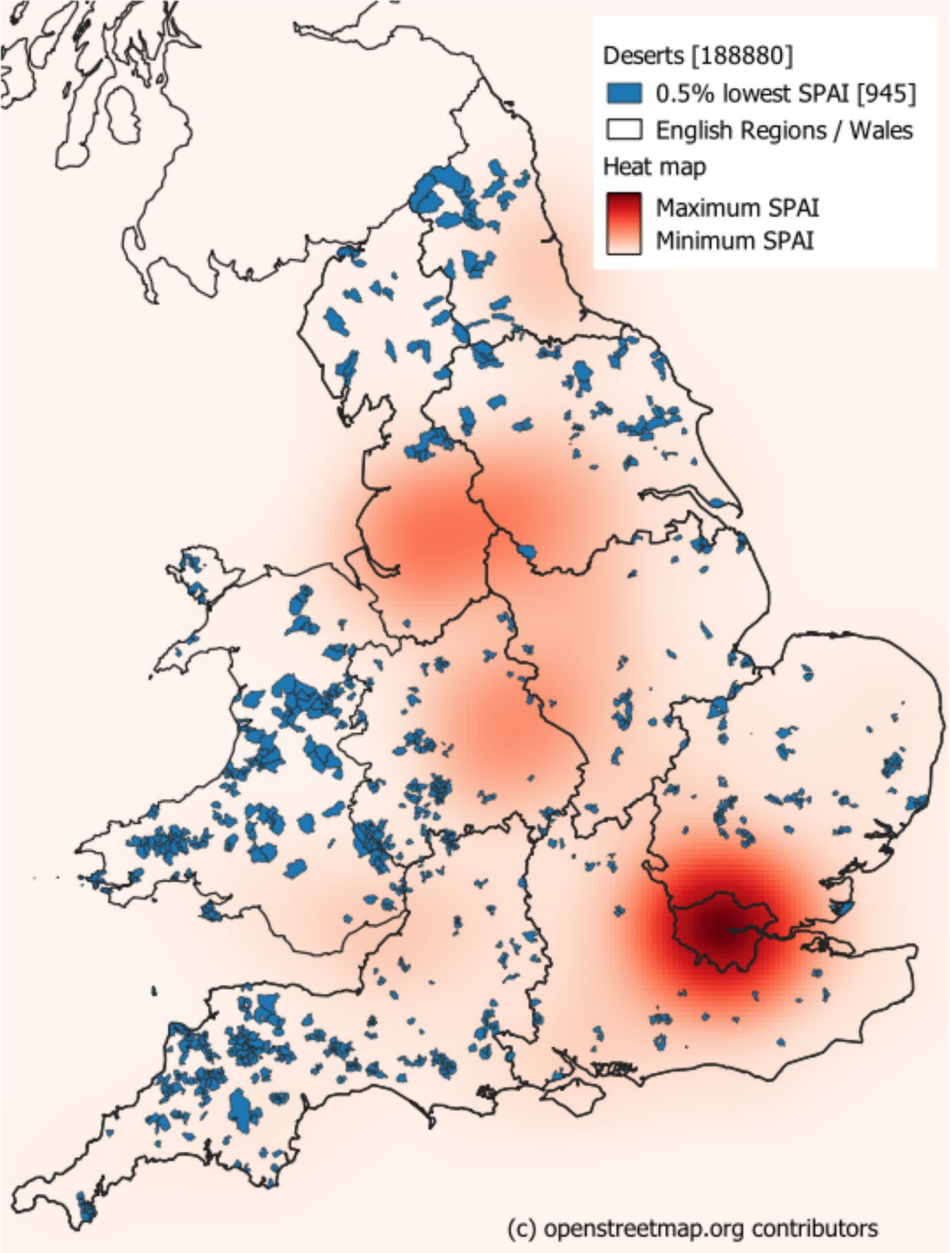
Locations of the 0.5% of Output Areas with lowest accessibility (blue) and a heat map of general accessibility (white to red)

Here a definition for a banking desert is adopted that defines such OAs as the 0.5% of OAs (945) with the lowest SPAI. Figure 1 shows where these OAs are located within England and Wales along with a base heat map showing areas with high accessibility. There is seen to be a large concentration of the low accessibility OAs in Wales and in western England along the Welsh borders, and also in the far south west of England. These locations are typified as being rural, with many towns and villages, but few cities. These areas also lack the density of the road network found in other areas of the country, with mountains, hills and moorland acting as barriers to travel. However there are similar rural locations where ATM deserts might be anticipated, e.g. the Lake District in the far North West of England, but are not so.

**Fig. 2:**
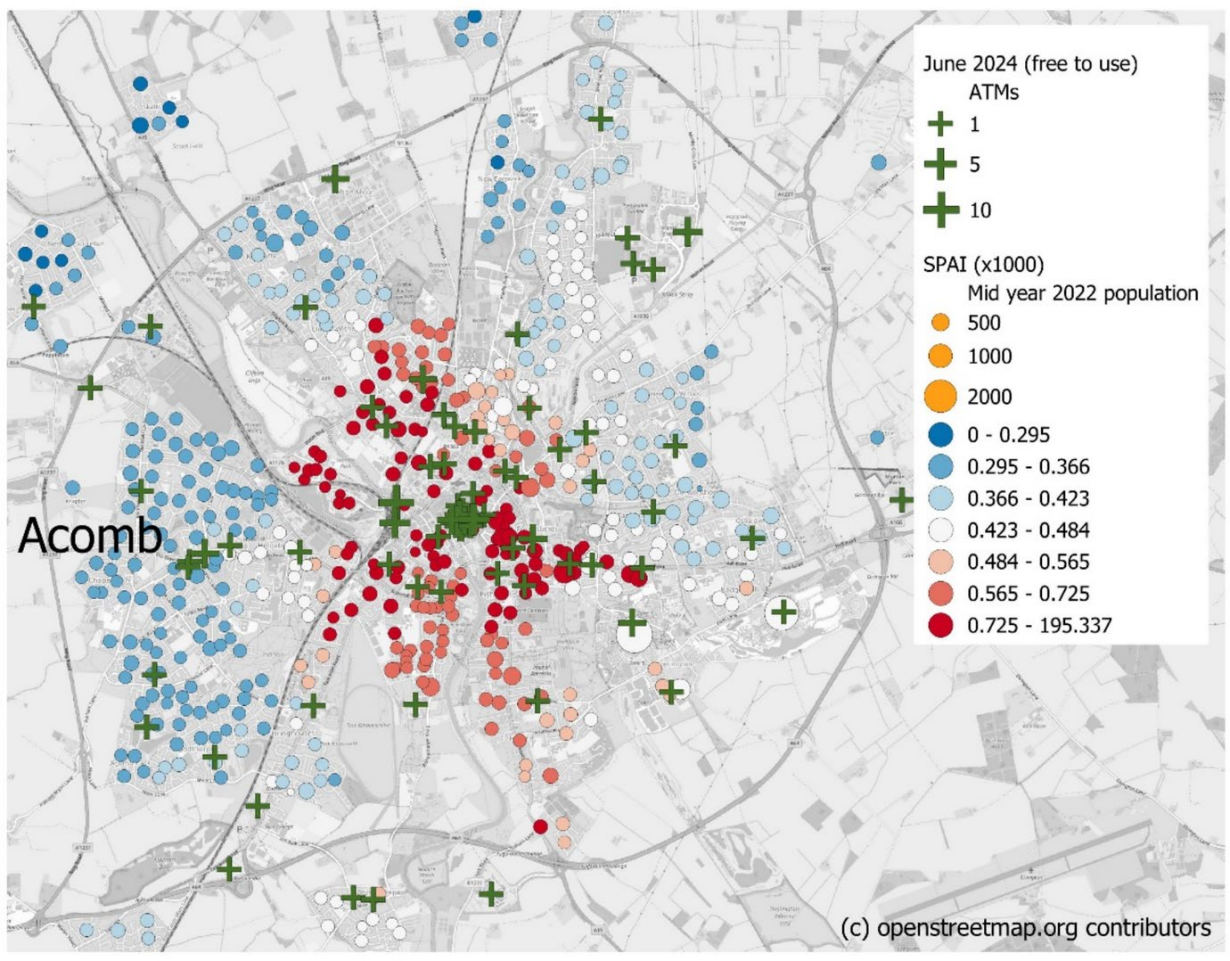
Accessibility for Output Areas in the City of York

Looking in more detail at a case study of the City of York, here there are no identified ATM deserts, but there is a difference in the accessibility to ATMs. This is shown in Fig. 2. There is in particular the suburb of Acomb to the west of the city centre that has low SPAI values, even though there is good provision of ATM locations within the suburb. However the presence of railway lines and the river Ouse provides barriers for travel to access some ATMs, particularly those in the city centre. This is similar to the findings of Leyshon et al., (2008) who identified the importance of physical barriers to access financial services, the presence of which can double the quoted journey distance to a location. These increased journey times lowers the SPAI. In contrast, OAs to the east of the river and the railway line, in spite of having fewer ATMs have fair to good accessibility, due to the absence of travel barriers to access the numerous city centre ATMs.

### Neighbourhood Typographies

The overall median SPAI and the median SPAI for various types of OAs are provided in Table 1. The reference situation is the middle column which is the network of free-to-use ATMs in June 2024. The left column is the situation three years earlier in June 2021, along with the percentage improvement with this historic SPAI. Comparing these two columns provides an assessment of how accessibility has changed over this period. Also supplementing the middle reference situation is the third rightmost column which is an assessment after including all ATMs in the June 2024 network, both those that are free-to-use and those that have a surcharge. Changes in these two columns indicates if certain types of OA ‘benefit’ more from the inclusion of the surcharging ATMs.

**Table 1:**
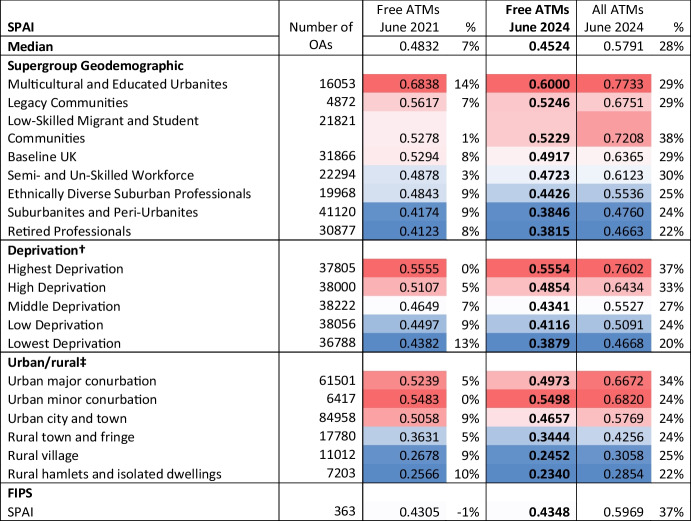
Median SPAI for various types of OA in England and Wales (scaled up by 1000) with % change relative to Free ATMs in June 2024 reference situation

Note †: No deprivation rank exists for 2021 OAs. Here each 2021 OA is given the deprivation of the 2011 Lower Super Output Area that contains the 2021 OA’s population weighted centroid.

‡: No urban/rural definition exists for 2021 OAs. Here each 2021 OA is given the urban/rural characteristic of the 2011 OA that contains the 2021 OA’s population weighted centroid.

For the Supergroup Geodemographic classification, the Baseline UK Supergroup appears in the middle of the rankings. Areas where the Supergroup is defined by a high degree of social challenge (low education, high unemployment, rented housing) tend to have higher SPAI and therefore better accessibility than Baseline UK (with the exception of the top Supergroup, Multicultural and Educated Urbanites, which is well educated). The three Supergroups with poorer accessibility than Baseline UK are more comfortable, with a population of owned or mortgaged larger properties, working in a profession or retired, and living outside urban cores. Looking over time, in all cases the SPAI was higher in June 2021 than June 2024, an indication of how the network has become less accessible. The inclusion of surcharge paying ATMs has, as expected, improved the accessibility across the board, but more so in the more challenged OAs mentioned above. Again looking at the differences, the Low-Skilled Migrant and Student Communities Supergroup is an exceptional case, with a particularly large increase in the SPAI after including these surcharge ATMs.

For deprivation there is a clear and consistent gradient in these results. The more deprived OAs have better accessibility to ATMs, and this is the case for both time periods and for both ATM networks.

For the urban/rural indicator in June 2024 there is not a uniform gradient with the degree of urbanisation. Urban minor conurbations have the highest accessibility, followed by the major urban conurbations. Thereafter the gradient goes with lower accessibility as the OA becomes more rural. The inclusion of surcharging ATMs again boosts accessibility, most noticeably for Urban major conurbations.

Finally there are those OAs identified as FIP neighbourhoods. In total there are 363 OAs that fall within a FIP neighbourhood, and the total mid-year 2022 estimate of the 16 and older population for these OAs is 92,085 people. These OAs typically have lower accessibility to the free ATM network than the median, however between June 2021 and June 2024 the accessibility for such OAs has improved slightly and the inclusion of surcharging ATMs boosts this accessibly considerably.

## Discussion

In this study an analysis of the ease of access to ATM locations has been conducted which builds on the earlier work of Langford et al., (2021) which also used an FCA technique to measure the accessibility of retail bank branches in Wales. It adds to the “*… increasing literature base which highlights the potential for approaches based around floating catchment area techniques that provide potentially more nuanced analyses of variations in access to a wide range of services*” (Langford et al., 2021). The work advances this understanding by firstly using enhancements to the E2SFCA with variable catchments (EV2SFCA) method that they used; it covers the whole of England and Wales and takes account of some edge issues with Scotland; it concentrates on the access to cash aspect of banking services, which widens the number of service locations considerably; uses a more recent 2022 mid-year population estimate; and finally it has incorporated some time series and network availability aspects.

Summarising the SPAI by various OA characteristics reveals a number of dynamics. The Supergroup with the highest accessibility (Multicultural and Educated Urbanites) is one typified by a population that is born outside the UK, with English not being their main language, well educated, single, and located in inner London and urban cities. It is this latter characteristic that most explains their good accessibility, with there being particular concentrations of ATMs in town and city centres. The other Supergroups that have better accessibility than Baseline UK are what could be termed economically and socially challenged OAs. They are populations that have low educational attainment, high unemployment and high likelihood of reported disabilities. Access to mainstream credit and financial products is difficult for such communities and their reliance of cash for every day purchases and budgeting has sustained the ATM network in such locations – to the extent that none of the OAs in these Supergroups feature as ATM deserts. The Supergroups with accessibility below that of Baseline UK are more affluent in nature and located in either sub-urban or rural locations. These differences are further emphasised when looking at the deprivation and rural/urban characteristics. More deprived and urban locations have better accessibility than affluent/sub-urban or rural locations (an issue studied by Griggs (2019)).

The accessibility for FIP OAs is low, a combination of the dual nature of this programme – to protect ATMs in both deprived and in rural OAs. Looking at the change from June 2021 it is clear that the FIP OAs have maintained their level of accessibility. If the SPAI for such OAs had shown a large decrease then this would have indicated that the programme had not achieved its main goal. On this evidence the program appears to be working. More generally the accessibility to ATMs has reduced over time, however this is not so much the case for the most deprived OAs, which experienced only a modest decrease in their accessibility, a reflection of the importance and utility of ATMs in such OAs and an especial desire by LINK to ensure such OAs have good access to ATMs..

The introduction of ATMs that have a surcharge improves the accessibility for all types of OA, but to different degrees. Accessibility improves the most in types of OAs were accessibility is already good. This suggest that these surcharging ATMs are not providing a service that supplements the network of free-to-use ATMs and they appear not to be exploiting a potential gap in the market, i.e. affluent, sub-urban and rural OAs.

### Policy

The SPAI has been mapped to identify locations where this accessibility is the lowest and these could be designated as OAs that are ATM deserts. Whilst some of these are single isolated locations, there are distinct clusters of OAs, particularly in Wales, the Welsh borders of England and in the South West of England. It is therefore most likely that any extra provision of ATMs strategically located within these clusters would have a maximal beneficial impact on residents’ access to cash. Indeed, the flexibility of the MHV3SFA allows various ‘what if’ scenarios to be explored to arrive at the best location for any new ATMs. This study shows that an “easy” option of converting all surcharging ATMs to be free to use would not be the best option, since the availability of such locations just re-enforces differences in accessibility rather than addressing them.

Looking beyond these potential deserts it is seen that even in urban locations there can exist pockets of poor access to ATMs. The case study of York illustrates that a joint consideration of both availability and ease of travel is required to get an accurate picture of accessibility. Thus changes to road and public transit network configurations, particularly in town and city centres have an impact here.

Another potentially useful output from this modelling process are ATMs in locations that are little needed or superfluous. Such ATMs may be identified as those whose supply-ratio (R_j_) is less than some threshold value, providing some evidence for locations where a rationalisation of the network would be least impactful. More significantly, superfluous ATMs are those that are not needed at all, i.e. they are not within the Q closest to any population centre; if they were removed from the data, the SPAIs would not change. However their numbers are small, with just 162 such superfluous ATMs locations in January 2024 and are mainly located in out of town shopping centres, motorway services stations and airports. For these types of ATM locations it is therefore important that information on actual usage would be needed before any decisions on their viability could be made.

### Limitations

Here for each journey between an ATM and an OA, only the quicker of two modes of travel are used, it may be insightful to model accessibility using just the quicker of say walking or transit, since the purchase and ownership of a motor vehicle may be financially prohibitive for some sections of society. This issue is discussed in Langford et al. (2016), who propose a many modes approach to calculating the journey times for SPAIs and also in Langford et al., (2022) where they matched branch opening times to transit schedules, which is of particular relevance here for ATMs located within business premises, but no so much for the ‘hole in the wall’ locations.

In this study and others (Langford et al., 2021) the demand population has been measured by the population aged 16 and older, an adult population. However the work by the Financial Conduct Authority (2021) has established that there are distinct typologies of people in the population where the need to access cash is more important than in the population as a whole. Thinking of the ‘Needs-based Users’, the ‘Functional Users’ and the ‘Older & Disengaged’, if neighbourhoods where these types were particularly concentrated can be identified, through an examination of neighbourhood traits, then the demand population can be further refined to reflect this differential in the need for cash. This would be better aligned with van Wee’s (2022) idea that some account is needed of the nature of the population in an accessibility calculation. If such an approach is taken then any optimisation of the ATM network could be biased toward helping such types to more easily access ATMs (much as the LINK FIP is attempting).

### Future Work

This method can be used to evaluate the impact on accessibility of changes in either the supply of ATMs or shifts in population. As further population projections become available then the demand, P_i_, can be adjusted and the model re-calculated. As the configuration of the ATM network, both free-to-use and surcharged changes then the impact can be evaluated (much as is done in Table 1). Beyond this representation of actual changes, hypothetical scenarios can be evaluated, e.g. switching some locations that currently have surcharged ATMs to be free-to-use. Table 1 begins to show that a wholesale conversation of surcharged ATMs to free-to-use ATMs might be ill-targeted and not be the best use of any resources.

Thinking about the journey times used in this study, whilst the use of road type specific speed limits to measure travel time is the most neutral assumption to adopt, it will not represent a worst case scenario, which is likely to occur in periods of peak traffic congestion. In extending this work, a travel time more grounded in actual network conditions may be useful. An extension to an even more multi-modal measure of travel, including additionally cycling and transit, would also be worth considering.

People do not always consider their access to cash from a residential perspective, but may use ATMs as part of their other day to day activities, such as shopping or at whilst at their place of work. Whilst small area worktime and daytime populations that might be used for this purpose are available from the 2021 Census there is a significant caveat that such counts were collected during the COVID-19 pandemic, when lock downs and requirements to work from home were in place.

## Data Availability

The data used in this study is available from the web sites referenced in the article. The authors do not have re-distribution rights for these data.

## Code Availability

The code used for the study is open source and referenced in the article.
